# A new index for characterizing micro-bead motion in a flow induced by ciliary beating: Part II, modeling

**DOI:** 10.1371/journal.pcbi.1005552

**Published:** 2017-07-14

**Authors:** Mathieu Bottier, Marta Peña Fernández, Gabriel Pelle, Daniel Isabey, Bruno Louis, James B. Grotberg, Marcel Filoche

**Affiliations:** 1 Eq. 13, Institut Mondor de Recherche Biomédicale, Inserm U955, Créteil, France; 2 Université Paris-Est, Faculté de médecine, Créteil, France; 3 CNRS ERL 7240, Créteil, France; 4 Department of Biomedical Engineering, University of Michigan, Ann Arbor, MI, USA; 5 Laboratoire de Physique de la Matière Condensée, Ecole Polytechnique, CNRS, Université Paris Saclay, 91128 Palaiseau Cedex, France; University of California Riverside, UNITED STATES

## Abstract

Mucociliary clearance is one of the major lines of defense of the human respiratory system. The mucus layer coating the airways is constantly moved along and out of the lung by the activity of motile cilia, expelling at the same time particles trapped in it. The efficiency of the cilia motion can experimentally be assessed by measuring the velocity of micro-beads traveling through the fluid surrounding the cilia. Here we present a mathematical model of the fluid flow and of the micro-beads motion. The coordinated movement of the ciliated edge is represented as a continuous envelope imposing a periodic moving velocity boundary condition on the surrounding fluid. Vanishing velocity and vanishing shear stress boundary conditions are applied to the fluid at a finite distance above the ciliated edge. The flow field is expanded in powers of the amplitude of the individual cilium movement. It is found that the continuous component of the horizontal velocity at the ciliated edge generates a 2D fluid velocity field with a parabolic profile in the vertical direction, in agreement with the experimental measurements. Conversely, we show than this model can be used to extract microscopic properties of the cilia motion by extrapolating the micro-bead velocity measurement at the ciliated edge. Finally, we derive from these measurements a scalar index providing a direct assessment of the cilia beating efficiency. This index can easily be measured in patients without any modification of the current clinical procedures.

## Author summary

Mucociliary clearance is the first line of defense mechanisms of the human airways. The mucus transporting debris, particles, microorganisms and pollutants is carried away by the coordinated motion of cilia beating at the surface of the airway epithelium. We present here a mathematical and numerical model aiming at defining a global index for assessing the efficiency of this beating. Numerical simulations show that the bead velocity parallel to the wall varies according a parabolic profile with the distance to the wall. The velocity extrapolated at the wall is demonstrated to be a measurement of the momentum transfer between cilia and the surrounding fluid. This model allows us to interpret experimental measurements performed in a companion article and to propose a universal index characterizing the beating efficiency, which can be extracted in the current clinical setting.

## Introduction

Mucociliary clearance is one of the major defense mechanisms of the respiratory airway system. The mucus layer coating the epithelial surface of the airways filters the inhaled air by trapping potentially harmful material (fungi, bacteria and other particles) [1–4]. This mucus layer is continuously carried away and out of the airways by the activity of motile cilia. Neighboring cilia beat in an organized manner with a small phase lag, their tips creating an undulating surface on top of the cilia layer which deforms in a wave-like fashion called the metachronal wave [5–7].

**Fig 1:**
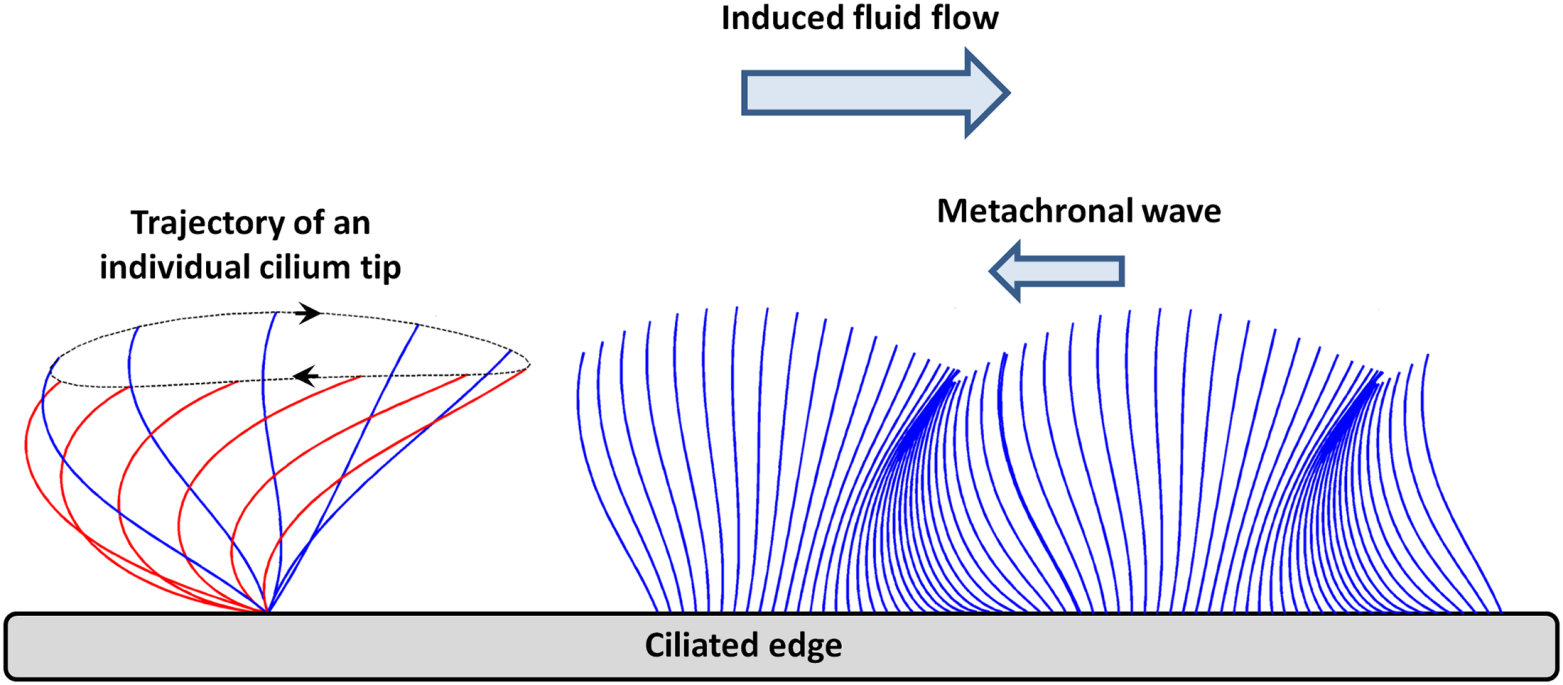
Schematic representation of the stroke of an individual cilium and the envelope model. (Left) Simplification of the stroke cycle as an ellipse. (Right) Representation of the envelope model covering the cilia layer and the propagation of the metachronal wave. (Inspired by Velez-Cordero et al. [23])

The beat pattern of an individual cilium displays a two-stroke effective-recovery motion [8]. During the effective stroke, cilia beat forwards and engage with the mucous layer, propelling it forward. In contrast, during the recovery stroke, they return to their initial position in the underlying periciliary fluid, minimizing thereby the drag on the mucus in the opposite direction (Fig 1, left). This asymmetry in the beat pattern is responsible for a net fluid flow in the direction of the effective stroke. In the airways, each mature ciliated cell may be covered with up to 200 cilia, with a surface density around 5–8 cilia/μm^2^ [6, 9]. A cilium, approximately 6 μm long and of diameter around 0.2 μm, beats 12 to 15 times per second, resulting in a velocity of the mucus layer of several mm/min [10].

This work intends to build a mathematical model of the experiments described in a companion paper [11], and to derive from this model an efficient and reliable method to assess the cilia beating efficiency in a clinical setting. In the experiments, ciliated cell clusters issued from nasal brushing are immersed in a cell survival medium devoid of mucus, pushing forward the medium as they beat. One has to stress here that the absence of mucus is an important ingredient of the experimental setting and the developed mathematical model. Polystyrene micro-beads are then used as massless tracers to visualize and quantify the fluid velocity field around the cilia. The theoretical and numerical work presented here therefore aim at a quantitative modeling of the ciliary beating, then of the fluid flow generated by it.

In the literature, two main types of ciliary beating models can be found: *discrete-cilia* models and *volume-force* models [12]. In *discrete-cilia* model, each cilium is modeled as a discrete body and its shape is parametrized along its stroke period [1, 13, 14]. *Discrete-cilia* models are themselves divided into two types: in the *prescribed beating* models, the cilium motion is imposed as an input to the simulation [15]; in the *couple-internal mechanics/fluid-structure interaction* models [16, 17], cilia motions originate from the coupling between the internal structure of cilia and the external viscous forces. In contrast, in *volume-force* models cilia are modeled through a phenomenological continuous force distribution, varying in space and time as the cilia beat [18–20]. In this second type of model, the envelope modeling approach accounts for the formation of metachronal waves above the cilia layer [21]. The cilia tips are seen from the fluid as a “wavy wall”, hereby ignoring the details of the sub-layer dynamics [22, 23] (see Fig 1, right).

Many studies have addressed experimentally [24–26] and numerically [27–31] the effect of fluid visco-elasticity on transport and locomotion. We want to stress here that in our model, no finite layer of viscous mucus sits on top of the cilia, since they are surrounded by a semi-infinite layer of watery fluid.

In the following, we first compute the wave envelope boundary condition from the cilia motions, based on the work of Ross [22]. We then derive the non linear equations for a fluid flow periodic in the direction of the metachronal wave. These equations are expanded in *ε*, which is the ratio of the cilium amplitude to the fluid layer thickness, then solved using a Fourier decomposition. The steady contribution to the flow field in the vertical direction above the cilia is shown to exhibit a parabolic profile, to a very good approximation. We finally show that measuring microbead velocities as a function of the distance to the ciliated edge enables us to compute a scalar index which accounts for the transfer of momentum between the cilia and the fluid, and therefore assesses the cilia beating efficiency.

## Materials and methods

We present here a two-dimensional model of cilia, fluid, and micro-beads motion.

### From the individual cilium motion to the metachronal wave

**Fig 2:**
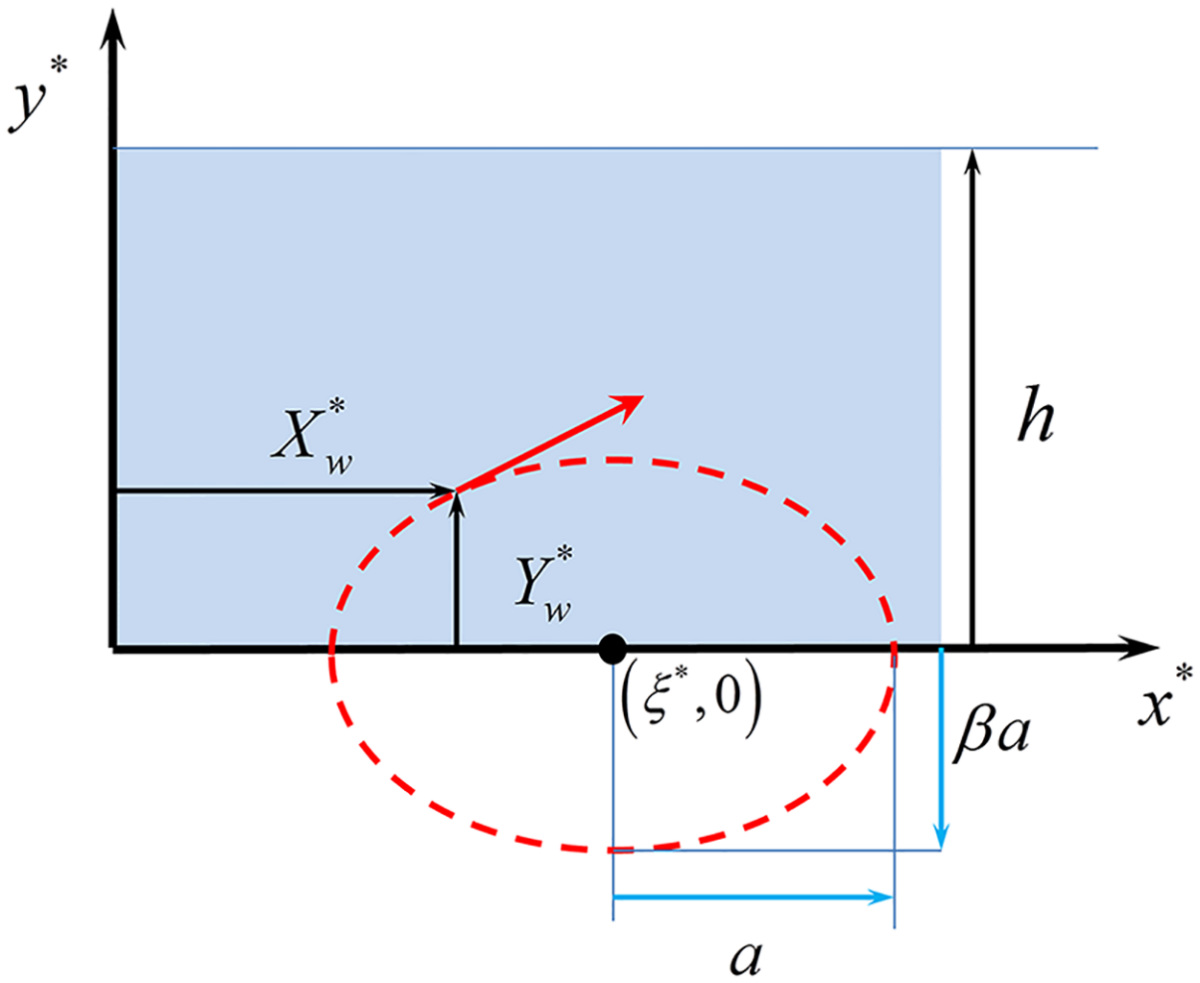
Schematic elliptic motion of an individual ciliary tip.

Each individual cilium is assumed to undergo a periodic motion in which its tip follows an elliptic trajectory, see Fig 1, left. Taking the limit of a continuous cilia distribution, the cilia array is simplified as an undulating surface that covers the cilia layer, ignoring the details of the sub-layer dynamics (Fig 1, right) [22, 23]. The x* axis is chosen parallel to the ciliated edge, each ciliary tip being located around y* = 0 on average (the ‘*’ notation stands for dimensioned quantities, and will be removed once we switch to dimensionless quantities). The tip of a cilium located at the horizontal coordinate ξ* is assumed to follow a periodic elliptic trajectory centered in (ξ*, 0) during each elementary beat (Fig 2). At time t*, the tip coordinates $(X_{w}^{*},Y_{w}^{*})$ are

$$\{\begin{array}{cc} X_{w}^{*} & =\xi^{*}-a\cos\left(\omega t^{*}\right)\\ Y_{w}^{*} & =\beta a\sin(\omega t^{*}) \end{array}$$ (1)

where *β* is the ellipse eccentricity, 2*a* is its major axis in the x* direction, and 2*βa* its minor axis in the y* direction. For *β* > 0, the tip orbits clockwise, while for *β* < 0, the tip orbits counterclockwise.

To reproduce the backwards traveling metachronal wave of wavelength λ, we introduce in the periodic motion of each cilium a phase shift $\frac{2\pi \xi^{*}}{\lambda }$ which linearly depends on the cilium position:

$$\{\begin{array}{cc} X_{w}^{*} & =\xi^{*}-a\cos\;(\omega t^{*}+\frac{2\pi \xi^{*}}{\lambda })\\ Y_{w}^{*} & =\beta a\sin\;(\omega t^{*}+\frac{2\pi \xi^{*}}{\lambda }) \end{array}$$ (2)

The corresponding wave frequency is *f* = 2*πω* and its speed is *c* = *f*λ. By setting the space and time units respectively to be *h* and *ω*^−1^, dimensionless parameters *ε* and *k*, and variables (*X*_*w*_, *Y*_*w*_) are introduced:

$$\varepsilon =\frac{a}{h}\;,\;k=\frac{2\pi h}{\lambda }\;,\;X_{w}=\frac{X_{w}^{*}}{h}\;,\;Y_{w}=\frac{Y_{w}^{*}}{h}\;,\;x=\frac{x^{*}}{h}\;,\;y=\frac{y^{*}}{h}\;,\;\xi =\frac{\xi^{*}}{h}\;,\;t=\omega t^{*}$$ (3)

The motion equations of each tip are thus rewritten in a dimensionless form:

$$\{\begin{array}{cc} X_{w}\left(\xi ,t\right) & =\xi -\varepsilon \cos(k\xi +t)\\ Y_{w}\left(\xi ,t\right) & =\beta \varepsilon \sin(k\xi +t) \end{array}$$ (4)

In the following, we assume that the ciliary beating amplitude is much smaller than the film thickness, i.e. *ε* ≪ 1. In the limit of continuously distributed cilia along the *x* axis, the envelope of their tip motions forms a continuous boundary that generates a forcing on the fluid layer above the cilia. Starting from Eq 4, we now express the position of a particle of this envelope (or wall) in the Eulerian frame of reference (*x*, *y*, *t*). A tip located at position *x* at time *t* corresponds to a cilium centered in (*ξ*, 0) such that:

x=ξ-εcos(kξ+t) (5)

This equation shows that (*x* − *ξ*) is of order *ε*. Therefore the vertical location of the cilia wall *y*_*w*_(*x*, *t*), which is also the y-coordinate of the corresponding cilium, *Y*_*w*_(*ξ*, *t*), can be developed around *ξ* = *x* using a Taylor expansion:

$$y_{w}\left(x,t\right)=Y_{w}\left(\xi ,t\right)=Y_{w}\left(x,t\right)+(\xi -x)\frac{\partial Y_{w}}{\partial \xi }\;{|}_{\xi =x}+\frac{{\left(\xi -x\right)}^{2}}{2}\frac{\partial^{2}Y_{w}}{\partial \xi^{2}}{|}_{\xi =x}+\cdots$$ (6)

Since both *Y*_*w*_ and (*ξ* − *x*) are first order in *ε* (Eqs 4 and 5), the expansion to the second order in *ε* of *y*_*w*_ is

$$y_{w}\left(x,t\right)=\varepsilon \beta \sin\left(kx+t\right)+\varepsilon^{2}\beta k\cos^{2}\left(kx+t\right)$$ (7)

Now that we have determined the location of the ciliated wall at time *t*, we can derive its velocity. The horizontal component of the wall velocity is obtained as the time derivative of the Lagrangian velocity of the tip at location *x* at time *t*, while its vertical component is calculated from the time derivative of the vertical coordinate of the wall at position *x* and time *t*:

$$\{\begin{array}{cc} u_{w}\left(x,t\right) & ={\left(\frac{\partial X_{w}}{\partial t}\right)}_{\xi }=\varepsilon \text{sin}\left(k\xi +t\right)\\ v_{w}\left(x,t\right) & =\frac{\partial y_{w}}{\partial t}=\varepsilon \beta \text{cos}\left(kx+t\right)-\varepsilon^{2}\beta k\text{sin}\left(2\left(kx+t\right)\right) \end{array}$$ (8)

The value of *ξ* to insert in the above horizontal velocity is given by Eq 5. Expanding the horizontal component of the wall velocity in Taylor series to the second order in *ε* yields

$$\begin{array}{ccc} u_{w}\left(x,t\right) & = & \frac{\partial X_{w}}{\partial t}\;{|}_{\xi =x}+\left(\xi -x\right)\frac{\partial u_{w}}{\partial \xi }{|}_{\xi =x}+\;\ldots \\ & = & \varepsilon \sin\left(kx+t\right)+\left[\varepsilon \cos\left(kx+t\right)\right]\left[\varepsilon k\cos\left(kx+t\right)\right]\\ & = & \varepsilon \sin\left(kx+t\right)+\varepsilon^{2}k\cos^{2}\left(kx+t\right) \end{array}$$ (9)

In summary, at location *x* in the Eulerian frame, the second order expansion in *ε* of the vertical position *y*_*w*_ and of the velocity vector (*u*_*w*_, *v*_*w*_) of the ciliated wall are:

$$y_{w}\left(x,t\right)=\varepsilon \beta \sin\theta +\frac{\varepsilon^{2}\beta k}{2}\;(1+\cos(2\theta ))$$ (10)

$$u_{w}\left(x,t\right)=\varepsilon \sin\theta +\frac{\varepsilon^{2}k}{2}\;(1+\cos(2\theta ))$$ (11)

$$v_{w}\left(x,t\right)=\varepsilon \beta \cos\theta -\varepsilon^{2}\beta k\sin\left(2\theta \right)$$ (12)

where *θ* = *kx* + *t* is the local phase of the metachronal wave. We now introduce the first and second orders of the *ε*-expansion of the wall velocity, called ${\vec{U}}_{w,1}=\left(u_{w,1},v_{w,1}\right)$ and ${\vec{U}}_{w,2}=\left(u_{w,2},v_{w,2}\right)$, respectively, defined such that:

$${\vec{U}}_{w}=\varepsilon \;{\vec{U}}_{w,1}+\frac{\varepsilon^{2}}{2}\;{\vec{U}}_{w,2}$$ (13)

In summary, the first and second orders in *ε* of the location and velocities of the cilia wall are:

$$\{\begin{array}{cc} y_{w,1}\left(\theta \right) & =\beta \sin\theta =\frac{\beta }{2i}e^{i\theta }-\frac{\beta }{2i}e^{-i\theta }\\ y_{w,2}\left(\theta \right) & =\beta k\;(1+\cos(2\theta ))=\beta k+\frac{\beta k}{2}e^{2i\theta }+\frac{\beta k}{2}e^{-2i\theta }\\ u_{w,1}\left(\theta \right) & =\sin\theta =\frac{1}{2i}e^{i\theta }-\frac{1}{2i}e^{-i\theta }\\ u_{w,2}\left(\theta \right) & =k\;(1+\cos(2\theta ))=k+\frac{k}{2}e^{2i\theta }+\frac{k}{2}e^{-2i\theta }\\ v_{w,1}\left(\theta \right) & =\beta \cos\theta =\frac{\beta }{2}e^{i\theta }+\frac{\beta }{2}e^{-i\theta }\\ v_{w,2}\left(\theta \right) & =-2\beta k\sin\left(2\theta \right)=i\beta k\;e^{2i\theta }-i\beta k\;e^{-2i\theta } \end{array}$$ (14)

We can observe therefore that all velocity terms at the ciliated wall are oscillatory, except one steady contribution to the horizontal velocity at the second order in *ε* that appears in *u*_*w*,2_. This contribution is proportional to *k* and will be the origin of the steady horizontal motion of the fluid above the cilia.

### Computing the flow field

We now compute the oscillatory flow field of a fluid of density *ρ* and viscosity *μ* in the channel comprised between *y* = *y*_*w*_(*x*, *t*) and *y* = *h* in the vertical direction. Due to the periodic nature of the forcing from the wall, we will consider periodic solution in the *x* direction. Given the normalization chosen in Eq 3, the normalization factors for velocity and pressure are *hω* and *μω*, respectively. Hence, the dimensionless Navier-Stokes equation reads

$$\alpha^{2}\;(\frac{\partial \vec{U}}{\partial t}+\left(\vec{U}\cdot \vec{\nabla }\right)\;\vec{U})=-\vec{\nabla }p+\Delta \vec{U}\;,$$ (15)

where $\vec{U}=\left(u,v\right)$ is the dimensionless velocity field, *p* is the dimensionless pressure, and *α* is the Womersley number defined by $\alpha^{2}=\frac{\rho h^{2}\omega }{\mu }$. In the limit *α* → 0, one recovers the classical stationary Stokes equation. In our case, typical values for the channel thickness and beating frequency are *h* = 50 *μ*m and *f* = 10 Hz. In water (*ρ* = 1 g.cm^−3^ and *μ* = 1 cP), the above expression leads to a Womersley number *α* ≈ 0.4, which means *α*^2^ of the order of 0.1.

#### Boundary conditions

Boundary conditions are periodic in the horizontal direction with a period equal to the wavelength of the metachronal wave (corresponding here to λ/*h* = 2*π*/*k*):

$$\vec{U}\;(\frac{2\pi }{k},y,t)=\vec{U}\left(0,y,t\right)$$ (16)

At the upper side of the domain (*y* = 1), one assumes vanishing vertical velocity and vanishing shear stress due to the presence of stagnant fluid above the channel:

$$v\left(x,1,t\right)=0\;\text{and}\;\frac{\partial u}{\partial y}{|}_{\left(x,1,t\right)}=0$$ (17)

Next to the cilia envelope (*y* = *y*_*w*_), we introduce a Robin-type boundary condition, which involves both the velocity and its normal derivative (Eq 18). The factor in front of the normal derivative, called *ϕ*, accounts for the partial momentum transfer between the wall and the fluid due to the non continuous coverage of the cilia. *ϕ* = 0 corresponds to a no slip boundary condition, while *ϕ* → +∞ corresponds to a vanishing shear stress at the wall (perfect sliding). This condition is analogous to that of a fluid flow next to a porous wall, where the presence of pores reduces the transfer of momentum between the wall and the fluid [32, 33]:

$$(\vec{U}-\phi \frac{\partial \vec{U}}{\partial y}){|}_{\left(x,y_{w},t\right)}={\vec{U}}_{w}\left(x,t\right)$$ (18)

If expressed in dimensional quantities, the parameter *ϕ* becomes a length ϕ* = *hϕ*. For a porous wall, this length is proportional to the square root of the permeability. In our case, it is interpreted as a characteristic sliding length and will be related to the cilia surface density.

Expanding the flow field $\vec{U}$ in powers of *ε*, $\vec{U}=\varepsilon \;{\vec{U}}_{1}+\frac{\varepsilon^{2}}{2}\;{\vec{U}}_{2}$, the boundary conditions can be expressed at all orders in *ε*. Since *y*_*w*_ is first order in *ε* (see Eq 10), the boundary condition can be expanded around *y* = 0 both for the velocity $\vec{U}$ and its normal derivative $\partial \vec{U}/\partial y$:

$$\begin{array}{ccc} \vec{U}\left(y_{w}\right) & = & \vec{U}\left(0\right)+y_{w}\frac{\partial \vec{U}}{\partial y}{|}_{y=0}+O\left(\varepsilon^{3}\right)\\ & = & \varepsilon {\vec{U}}_{1}\left(0\right)+\frac{\varepsilon^{2}}{2}{\vec{U}}_{2}\left(0\right)+[\varepsilon y_{w,1}+\frac{\varepsilon^{2}}{2}y_{w,2}]\;[\varepsilon \frac{\partial {\vec{U}}_{1}}{\partial y}{|}_{0}+\frac{\varepsilon^{2}}{2}\frac{\partial {\vec{U}}_{2}}{\partial y}{|}_{0}]+O\left(\varepsilon^{3}\right)\\ & = & \varepsilon \;{\vec{U}}_{1}\left(0\right)+\frac{\varepsilon^{2}}{2}\;[{\vec{U}}_{2}\left(0\right)+2y_{w,1}\frac{\partial {\vec{U}}_{1}}{\partial y}]+O\left(\varepsilon^{3}\right) \end{array}$$ (19)

$$\begin{array}{ccc} \frac{\partial \vec{U}}{\partial y}{|}_{y_{w}} & = & \frac{\partial \vec{U}}{\partial y}{|}_{0}+y_{w}\frac{\partial^{2}\vec{U}}{\partial y^{2}}{|}_{0}+O\left(\varepsilon^{3}\right)\\ & = & \varepsilon \frac{\partial {\vec{U}}_{1}}{\partial y}{|}_{0}+\frac{\varepsilon^{2}}{2}\frac{\partial {\vec{U}}_{2}}{\partial y}{|}_{0}+[\varepsilon y_{w,1}+\frac{\varepsilon^{2}}{2}y_{w,2}]\;[\varepsilon \frac{\partial^{2}{\vec{U}}_{1}}{\partial y^{2}}{|}_{0}+\frac{\varepsilon^{2}}{2}\frac{\partial^{2}{\vec{U}}_{2}}{\partial y^{2}}{|}_{0}]+O\left(\varepsilon^{3}\right)\\ & = & \varepsilon \;\frac{\partial {\vec{U}}_{1}}{\partial y}{|}_{0}+\frac{\varepsilon^{2}}{2}\;[\frac{\partial {\vec{U}}_{2}}{\partial y}{|}_{0}+2y_{w,1}\frac{\partial^{2}{\vec{U}}_{1}}{\partial y^{2}}{|}_{0}]+O\left(\varepsilon^{3}\right) \end{array}$$ (20)

Inserting Eqs 19 and 20 into Eq 18 finally gives the first two orders of the *ε*-expansion of the boundary condition at the ciliated wall:

$${\vec{U}}_{1}\left(0\right)-\phi \frac{\partial {\vec{U}}_{1}}{\partial y}{|}_{0}={\vec{U}}_{w,1}$$ (21)

$${\vec{U}}_{2}\left(0\right)-\phi \frac{\partial {\vec{U}}_{2}}{\partial y}{|}_{0}={\vec{U}}_{w,2}-2y_{w,1}(\frac{\partial {\vec{U}}_{1}}{\partial y}{|}_{0}-\phi \frac{\partial^{2}{\vec{U}}_{1}}{\partial y^{2}}{|}_{0})$$ (22)

### The stream function

Since the flow is two-dimensional and incompressible, a natural formulation of the problem is obtained by introducing the stream function *ψ* such that:

$$u=\frac{\partial \psi }{\partial y}\;\text{and}\;v=-\frac{\partial \psi }{\partial x}$$ (23)

Such a solution automatically fulfills the continuity equation $\text{div}(\vec{U})=0$. The dimensionless Navier-Stokes equation now reads:

$$\{\begin{array}{cc} \alpha^{2}(\psi_{yt}+\psi_{y}\psi_{yx}-\psi_{x}\psi_{yy}) & =-p_{x}+\psi_{yxx}+\psi_{yyy}\\ \alpha^{2}(-\psi_{xt}+\psi_{y}\psi_{xx}+\psi_{x}\psi_{xy}) & =-p_{y}-\psi_{xxx}-\psi_{xyy} \end{array}$$ (24)

We derive a Partial Differential Equation (PDE) in *ψ* only by taking the y-derivative of the first equation and subtracting it to it the x-derivative of the second equation:

$$\alpha^{2}\;\{\psi_{xxt}+\psi_{yyt}+\psi_{y}\psi_{yyx}-\psi_{x}\psi_{yyy}+\psi_{y}\psi_{xxx}-\psi_{x}\psi_{xxy}\}=\psi_{yyyy}+2\psi_{xx}\psi_{yy}+\psi_{xxxx}$$ (25)

This equation can finally be rewritten in the following compact form:

$$\alpha^{2}\;\{\Delta \psi_{t}+\psi_{y}\Delta \psi_{x}-\psi_{x}\Delta \psi_{y}\}=\Delta^{2}\psi$$ (26)

#### Asymptotic expansion of the stream function

We now expand the stream function to the second order in *ε*, as it was done for the flow field:

$$\psi =\varepsilon \psi_{1}+\frac{\varepsilon^{2}}{2}\psi_{2}$$ (27)

The zero order term *ψ*_0_ vanishes since the fluid is set into motion only by the cilia beating (*ε* > 0). The two orders in *ε* are solved in sequence. The first order of Eq 26 is:

$$\alpha^{2}\Delta \psi_{1,t}=\Delta^{2}\psi_{1}\;$$ (28)

while the second order is:

$$\alpha^{2}\;\{\Delta \psi_{2,t}+2\psi_{1,y}\Delta \psi_{1,x}-2\psi_{1,x}\Delta \psi_{1,y}\}=\Delta^{2}\psi_{2}$$ (29)

The boundary condition at the lower wall (the cilia envelope) couples the first and second orders *ψ*_1_ and *ψ*_2_ through the Robin condition of Eq 18. The two velocity components and the two orders in *ε* translate into 4 equations at the wall boundary:

$$\psi_{1,y}{|}_{\left(x,0\right)}-\phi \;\psi_{1,yy}=\sin\theta$$ (30)

$$\psi_{1,x}{|}_{\left(x,0\right)}-\phi \;\psi_{1,xy}=-\beta \cos\theta$$ (31)

$$\psi_{2,y}{|}_{\left(x,0\right)}-\phi \;\psi_{2,yy}=k(1+\cos(2\theta ))-2\beta \sin\theta \;(\psi_{1,yy}-\phi \;\psi_{1,yyy})$$ (32)

$$\psi_{2,x}{|}_{\left(x,0\right)}-\phi \;\psi_{2,xy}=2\beta k\sin\left(2\theta \right)-2\beta \sin\theta \;(\psi_{1,xy}-\phi \;\psi_{1,xyy})$$ (33)

The stream function can thus be computed in first approximation as if the domain were a straight channel comprised between *y* = 0 and *y* = 1.

#### First order of the stream function

*ψ*_1_(*x*, *y*, *t*) is a periodic function of period 2*π*/*k* along *x* that solves the following system:

$$\{\begin{array}{cc} \Delta^{2}\psi_{1}-\alpha^{2}\Delta \psi_{1,t}=0\\ \psi_{1,y}-\phi \;\psi_{1,yy}=\sin\theta \; & \text{at}\;y=0\\ \psi_{1,x}-\phi \;\psi_{1,xy}=-\beta \cos\theta \; & \text{at}\;y=0\\ \psi_{1,yy}=0\; & \text{at}\;y=1\\ \psi_{1,x}=0\; & \text{at}\;y=1 \end{array}$$ (34)

We expand *ψ*_1_ in Fourier series along the *x* direction, each term corresponding to a wave traveling forward or backward at dimensionless speed 1.

$$\psi_{1}\left(x,y,t\right)=\sum_{n=-\infty }^{+\infty }\;a_{n}\left(y\right)\;e^{in(kx+t)}$$ (35)

Due to the linearity of the PDE in Eq 34, each term of this Fourier series satisfies the same equation, which implies:

$$(a_{n}^{\prime \prime \prime \prime }-2k^{2}n^{2}a_{n}^{\prime \prime }+k^{4}n^{4}a_{n})-in\alpha^{2}\;(a_{n}^{\prime \prime }-k^{2}n^{2}a_{n})=0\;.$$ (36)

this linear ODE is solved using its characteristic equation:

$$\delta^{4}-(2k^{2}n^{2}+in\alpha^{2})\;\delta^{2}+k^{2}n^{2}\;(k^{2}n^{2}+in\alpha^{2})=0\;$$ (37)

whose 4 solutions are:

$$\delta =\pm k|n|\;\text{and}\;\delta =\pm \gamma_{n}\;\text{with}\;\gamma_{n}=\sqrt{k^{2}n^{2}+in\alpha^{2}}$$ (38)

The coefficients of the Fourier decomposition of *ψ*_1_ are determined by the boundary conditions. In real experiments carried out on fluid flow next to ciliated edges [11], the value of *k* is much larger than 1 (which means that the channel width along y is much larger than the metachronal wavelength). This enables us to translate the boundary condition at *y* = 1 (the last two of Eq 34) into the cancellation of the coefficients attached to the roots with positive real part in Eq 37. Finally, since the only non homogeneous terms appearing in these boundary conditions are in *e*^*iθ*^ and *e*^−*iθ*^ (sin *θ* and cos *θ*), we only retain the coefficients corresponding to *n* = 1 and *n* = −1, which leads to the following expression for *ψ*_1_:

$$\psi_{1}\left(x,y,t\right)=[A_{1}e^{-ky}+B_{1}e^{-\gamma_{1}y}]\;e^{i\theta }+[A_{-1}e^{-ky}+B_{-1}e^{-\gamma_{-1}y}]\;e^{-i\theta }$$ (39)

Since $\gamma_{-1}={\bar{\gamma }}_{1}$, imposing that *ψ*_1_ is real valued implies that the coefficients also satisfy $A_{-1}={\bar{A}}_{1}$ and $B_{-1}={\bar{B}}_{1}$. Therefore, *ψ*_1_ can be expressed as:

$$\psi_{1}\left(x,y,t\right)=[A_{1}e^{-ky}+B_{1}e^{-\gamma_{1}y}]\;e^{i\theta }+c.c.\;\;,$$ (40)

where c.c. stands for complex conjugate. The flow field at first order in *ε* is then:

$$\{\begin{array}{c} u_{1}=\psi_{1,y}=-2\;\text{Re}\{[kA_{1}e^{-ky}+\gamma_{1}B_{1}e^{-\gamma_{1}y}]\;e^{i\theta }\}\\ v_{1}=-\psi_{1,x}=-2k\;\text{Im}\{[A_{1}e^{-ky}+B_{1}e^{-\gamma_{1}y}]\;e^{i\theta }\} \end{array}$$ (41)

*A*_1_ and *B*_1_ are determined from the two boundary conditions at *y* = 0 in Eq (34):

$$\{\begin{array}{cc} -kA_{1}-\gamma_{1}B_{1}-\phi \;(k^{2}A_{1}+\gamma_{1}^{2}B_{1}) & =\frac{1}{2i}\\ ik[A_{1}+B_{1}-\phi \;(-kA_{1}-\gamma_{1}B_{1})] & =-\frac{\beta }{2} \end{array}$$ (42)

which finally gives:

$$A_{1}=\frac{i}{2(1+k\phi )}\;(\frac{\frac{\beta \gamma_{1}}{k}-1}{\gamma_{1}-k})\;\text{and}\;B_{1}=\frac{i}{2(1+\gamma_{1}\phi )}\;(\frac{1-\beta }{\gamma_{1}-k})$$ (43)

#### Second order of the stream function: The steady contribution

*ψ*_2_(*x*, *y*, *t*), the second order in *ε* of the stream function, is a periodic function of period 2*π*/*k* along *x* that solves the following system:

$$\{\begin{array}{cc} \Delta^{2}\psi_{2}-\alpha^{2}\Delta \psi_{2,t}=2\alpha^{2}\{\psi_{1,y}\Delta \psi_{1,x}-\psi_{1,x}\Delta \psi_{1,y}\}\\ \psi_{2,y}-\phi \;\psi_{2,yy}=k(1+\cos(2\theta ))-2\beta \sin\theta \;\psi_{1,yy}+2\beta \phi \sin\theta \;\psi_{1,yyy}\; & \text{at}\;y=0\\ \psi_{2,x}-\phi \;\psi_{2,xy}=2\beta k\sin\left(2\theta \right)-2\beta \sin\theta \;\psi_{1,xy}+2\beta \phi \sin\theta \;\psi_{1,xyy}\; & \text{at}\;y=0 \end{array}$$ (44)

Since the first order *ψ*_1_ of the stream function contains only terms in *e*^*iθ*^ and *e*^−*iθ*^, as seen in the previous section, a rapid analysis of the above boundary conditions shows that the second order *ψ*_2_ will only contain terms either constant or proportional to *e*^2*iθ*^ and *e*^−2*iθ*^. We are interested here only in the constant term, which corresponds to the first non vanishing contribution to the steady flow field in the *ε*-expansion. From Eq 29, we know that the steady part of *ψ*_2_, here called $\psi_{2}^{\left(s\right)}$, solves the following equation

$$\Delta^{2}\psi_{2}^{\left(s\right)}=2\alpha^{2}\;\;{\left\{\psi_{1,y}\Delta \psi_{1,x}-\psi_{1,x}\Delta \psi_{1,y}\right\}}^{\left(s\right)}$$ (45)

where the superscript ‘(s)’ on the right hand side stands for the steady part. More precisely, the bracketed term can be rewritten as:

$$\begin{array}{ccc} \psi_{1,y}\Delta \psi_{1,x}-\psi_{1,x}\Delta \psi_{1,y} & = & \psi_{1,y}\left(\psi_{1,xxx}+\psi_{1,xyy}\right)-\psi_{1,x}\left(\psi_{1,xxy}+\psi_{1,yyy}\right)\\ & = & \left(\psi_{1,y}\psi_{1,xyy}-\psi_{1,x}\psi_{1,yyy}\right)+\left(\psi_{1,y}\psi_{1,xxx}-\psi_{1,x}\psi_{1,xxy}\right)\\ & = & {\left(\psi_{1,y}\psi_{1,xy}-\psi_{1,x}\psi_{1,yy}\right)}_{y}+{\left(\psi_{1,y}\psi_{1,xx}-\psi_{1,x}\psi_{1,xy}\right)}_{x} \end{array}$$ (46)

Let us examine the *x*-dependency of the parentheses appearing in the last line. All products contain terms that are either constant or oscillating in *x*. Therefore the contribution of the second parenthesis to the steady stream function can only be constant. Its *x*-derivative then vanishes and can be removed from the equation satisfied by $\psi_{2}^{\left(s\right)}$, leaving only the first parenthesis:

$$\psi_{2,yyyy}^{\left(s\right)}=2\alpha^{2}\;{\left(\psi_{1,y}\psi_{1,yx}-\psi_{1,x}\psi_{1,yy}\right)}_{y}^{\left(s\right)}$$ (47)

Integrating along *y* yields:

$$\psi_{2,yyy}^{\left(s\right)}=2\alpha^{2}\;{\left(\psi_{1,y}\psi_{1,yx}-\psi_{1,x}\psi_{1,yy}\right)}^{\left(s\right)}+2A_{2}$$ (48)

where 2*A*_2_ is an integration constant. Note that *ε*^2^/2 × 2*A*_2_ = *ε*^2^ *A*_2_ can actually be interpreted as the steady component of the pressure gradient $p_{x}^{\left(s\right)}$ which appears in the right hand side of the Navier-Stokes equation (Eq 15). The model therefore predicts a constant pressure gradient along *x*. From the previous section, we know that *ψ*_1_ can be written as:

$$\psi_{1}\left(x,y,t\right)=a_{1}\left(y\right)\;e^{i\theta }+c.c.\;\text{with}\;a_{1}\left(y\right)=A_{1}e^{-ky}+B_{1}e^{-\gamma_{1}y}$$ (49)

Inserting this expression of *ψ*_1_ into the right hand side parenthesis of Eq 48 leaves:

$${\left(\psi_{1,y}\psi_{1,yx}-\psi_{1,x}\psi_{1,yy}\right)}^{\left(s\right)}=-ik|{a^{\prime }}_{1}{|}^{2}\;-\;ika_{1}{{\bar{a}}^{\prime \prime }}_{1}+c.c.=2k\;\text{Im}(a_{1}{{\bar{a}}^{\prime \prime }}_{1})$$ (50)

(The first term of the above right hand side being imaginary, its contribution vanishes when adding its complex conjugate). Eq 48 and the first boundary condition of Eq 44 thus provide the following simpler system for $\psi_{2}^{\left(s\right)}$ (the second boundary condition deals with *x*-derivatives that vanish in the steady flow due to the translation invariance of the problem):

$$\{\begin{array}{c} \psi_{2,yyy}^{\left(s\right)}=4\alpha^{2}k\;\text{Im}\left(a_{1}{\bar{a}}_{1}^{\prime \prime }\right)+2A_{2}\\ \psi_{2,y}^{\left(s\right)}\left(x,0\right)-\phi \;\psi_{2,yy}^{\left(s\right)}\left(x,0\right)=k+C \end{array}$$ (51)

where *C* stands for the constant contribution of −2*β* sin *θ* (*ψ*_1, *yy*_ − *ϕ* *ψ*_1, *yyy*_)|_*y* = 0_. This constant *C* is determined using the expression of *ψ*_1_ in Eq 49:

$$\psi_{1,yy}-\phi \;\psi_{1,yyy}=\left(a_{1}^{\prime \prime }-\phi \;a_{1}^{\prime \prime \prime }\right)\;e^{i\theta }+c.c.$$ (52)

Therefore the constant contribution *C* can be expressed from the constants *A*_1_ and *B*_1_ computed in the previous section:

$$C=2\beta \;\text{Im}(a_{1}^{\prime \prime }\left(0\right)-\phi \;a_{1}^{\prime \prime \prime }\left(0\right))=2\beta \;\text{Im}(k^{2}A_{1}+\gamma_{1}^{2}B_{1}+k^{3}\phi A_{1}+\gamma_{1}^{3}\phi B_{1})$$ (53)

Using the values of *A*_1_ and *B*_1_ obtained in the previous section gives immediately:

$$C=2\beta \;\text{Im}\;[\frac{ik^{2}}{2}\;(\frac{\frac{\beta \gamma_{1}}{k}-1}{\gamma_{1}-k})+\frac{i\gamma_{1}^{2}}{2}\;(\frac{1-\beta }{\gamma_{1}-k})]=\beta k+\beta \left(1-\beta \right)\;\text{Re}\left(\gamma_{1}\right)$$ (54)

We now turn to Im($a_{1}{\bar{a}}_{1}^{\prime \prime }$) in order to determine $\psi_{2}^{\left(s\right)}$. Using Eq 40, this term can be rewritten:

$$\begin{array}{ccc} \text{Im}\left(a_{1}{\bar{a}}_{1}^{\prime \prime }\right) & = & \text{Im}\;\{(A_{1}e^{-ky}+B_{1}e^{-\gamma_{1}y})(k^{2}{\bar{A}}_{1}e^{-ky}+{\bar{\gamma }}_{1}^{2}\bar{B_{1}}e^{-\bar{\gamma_{1}}y})\}\\ & = & \text{Im}\;\{{\bar{\gamma }}_{1}^{2}A_{1}\bar{B_{1}}e^{-(k+{\bar{\gamma }}_{1})y}+k^{2}{\bar{A}}_{1}B_{1}e^{-(k+\gamma_{1})y}+{\bar{\gamma }}_{1}^{2}B_{1}\bar{B_{1}}e^{-(\gamma_{1}+{\bar{\gamma }}_{1})y}\} \end{array}$$ (55)

Using the relation ${\bar{\gamma }}_{1}^{2}=k^{2}-i\alpha^{2}$ allows us to simplify the above expression to:

$$\begin{array}{ccc} \text{Im}\left(a_{1}{\bar{a}}_{1}^{\prime \prime }\right) & = & \text{Im}\;\{-i\alpha^{2}A_{1}\bar{B_{1}}e^{-(k+{\bar{\gamma }}_{1})y}-i\alpha^{2}{\left|B_{1}\right|}^{2}e^{-(\gamma_{1}+{\bar{\gamma }}_{1})y}\}\\ & = & -\alpha^{2}\;[\text{Re}(A_{1}\bar{B_{1}}e^{-(k+{\bar{\gamma }}_{1})y})+{\left|B_{1}\right|}^{2}e^{-(\gamma_{1}+{\bar{\gamma }}_{1})y}] \end{array}$$ (56)

This expression is now inserted into the first equation of Eq 51, which gives after 2 integrations:

$$\psi_{2,y}^{\left(s\right)}=A_{2}\;y^{2}+B_{2}\;y+C_{2}-4\alpha^{4}k\;\;\left[\text{Re}\;\;\left(A_{1}{\bar{B}}_{1}\;\frac{e^{-\left(k+{\bar{\gamma }}_{1}\right)y}}{{\left(k+{\bar{\gamma }}_{1}\right)}^{2}}\right)+{\left|B_{1}\right|}^{2}\;\frac{e^{-\left(\gamma_{1}+{\bar{\gamma }}_{1}\right)y}}{{\left(\gamma_{1}+{\bar{\gamma }}_{1}\right)}^{2}}\right],$$ (57)

where *B*_2_ and *C*_2_ are integration constants. We observe here that the steady contribution to the horizontal fluid velocity appears as the sum of one parabolic and two exponential profiles. The parabolic profile depends on integration constants *A*_2_, *B*_2_, and *C*_2_ that are determined using the boundaries conditions. Velocity and shear stress vanish at *y* = 1, so that the fluid in the flowing layer matches the stagnant fluid above. The boundary conditions take the following form:

$$\{\begin{array}{cc} \psi_{2,y}^{\left(s\right)}\left(1\right) & =0=A_{2}+B_{2}+C_{2}+G_{1}\\ \psi_{2,yy}^{\left(s\right)}\left(1\right) & =0=2A_{2}+B_{2}+G_{2}\\ \psi_{2,y}^{\left(s\right)}\left(0\right)-\phi \;\psi_{2,yy}^{\left(s\right)}\left(0\right) & =k+C=C_{2}-\phi B_{2}+G_{3}+k+C \end{array}$$ (58)

where *G*_1_, *G*_2_, and *G*_3_ are defined such that:

$$\{\begin{array}{ccc} \frac{G_{1}}{4\alpha^{4}k} & = & -\text{Re}\;\left(A_{1}{\bar{B}}_{1}\;\frac{e^{-(k+{\bar{\gamma }}_{1})}}{{\left(k+{\bar{\gamma }}_{1}\right)}^{2}}\right)-|B_{1}{|}^{2}\;\frac{e^{-(\gamma_{1}+{\bar{\gamma }}_{1})}}{{\left(\gamma_{1}+{\bar{\gamma }}_{1}\right)}^{2}}\\ \frac{G_{2}}{4\alpha^{4}k} & = & \text{Re}\;\left(A_{1}{\bar{B}}_{1}\;\frac{e^{-(k+{\bar{\gamma }}_{1})}}{\left(k+{\bar{\gamma }}_{1}\right)}\right)+|B_{1}{|}^{2}\;\frac{e^{-(\gamma_{1}+{\bar{\gamma }}_{1})}}{\left(\gamma_{1}+{\bar{\gamma }}_{1}\right)}\\ \frac{G_{3}+k+C}{4\alpha^{4}k} & = & -\text{Re}\;\left(\frac{A_{1}{\bar{B}}_{1}}{{\left(k+{\bar{\gamma }}_{1}\right)}^{2}}\right)-\phi \;\text{Re}\;\left(\frac{A_{1}{\bar{B}}_{1}}{\left(k+{\bar{\gamma }}_{1}\right)}\right)-\frac{|B_{1}{|}^{2}}{{\left(\gamma_{1}+{\bar{\gamma }}_{1}\right)}^{2}}-\phi \frac{|B_{1}{|}^{2}}{\left(\gamma_{1}+{\bar{\gamma }}_{1}\right)} \end{array}$$ (59)

The coefficients of the steady parabolic profile are finally:

$$\{\begin{array}{cc} A_{2} & =\frac{G_{1}-\left(1+\phi \right)G_{2}-G_{3}}{1+2\phi }\\ B_{2} & =\frac{-2G_{1}+G_{2}+2G_{3}}{1+2\phi }\\ C_{2} & =\frac{-2\phi \;G_{1}+\phi \;G_{2}-G_{3}}{1+2\phi } \end{array}$$ (60)

In summary, the velocity field of the fluid flow can be expanded in powers of *ε*. The first non vanishing order in *ε* for the oscillatory part of the fluid flow is of order 1 while at the second order, a steady contribution appears. This steady flow is the one responsible for the micro-bead motion observed in the experiments. It has essentially a parabolic profile whose coefficients are directly determined from the values of the quantities *k*, *α*, *β*, and *ϕ*, which are in turn deduced from the fundamental parameters of the cilia motion and the surrounding fluid (frequency, amplitude, density, viscosity). In the experiments, fitting the measured micro-bead velocities with a parabolic profile will allow us to extract quantities that are not easily measurable, such as the cilium amplitude *a* or the transfer parameter *ϕ*.

#### The steady velocity profile

In experiments carried out on real samples, the values of *k* are larger than 10 (in most cases much larger), and *α*^2^ is about 0.1. We can therefore safely assume that *e*^−*k*^ ≪ 1 and *α* ≪ 1. In this situation, *G*_1_ and *G*_2_ are negligible compared to *G*_3_, and the coefficients of the parabolic profile are:

$$A_{2}=-\frac{G_{3}}{1+2\phi }\;,\;B_{2}=\frac{2G_{3}}{1+2\phi }\;,\;C_{2}=-\frac{G_{3}}{1+2\phi }$$ (61)

This leads to the following steady flow:

$$u\left(y\right)=-\frac{\varepsilon^{2}}{2}\;(\frac{G_{3}}{1+2\phi }\;y^{2}+\frac{2G_{3}}{1+2\phi }\;y-\frac{G_{3}}{1+2\phi })=-\frac{\varepsilon^{2}}{2}\frac{G_{3}}{1+2\phi }\;{\left(y-1\right)}^{2}$$ (62)

One recovers here a parabolic profile with vanishing velocity and vanishing shear stress at *y* = 1. Moreover, from Eq 59, the term proportional to 4*α*^4^*k* in *G*_3_ can be neglected, leading to:

$$G_{3}\approx -k-C=-k-\beta k-\beta \left(1-\beta \right)\;\text{Re}\left(\gamma_{1}\right)\approx \left(\beta^{2}-2\beta -1\right)k$$ (63)

The dimensionless extrapolated velocity at the ciliated wall in this case is then:

$$u\left(0\right)=\frac{\varepsilon^{2}}{2}\;(\frac{1+2\beta -\beta^{2}}{1+2\phi })k=\frac{\varepsilon^{2}}{2}\;(\frac{2-{\left(\beta -1\right)}^{2}}{1+2\phi })\;k$$ (64)

One can see here that the extrapolation of the flow velocity (hence of the microbead) at the ciliated wall depends on *β*, *ϕ*, and *k*. It has a maximum value, *ε*^2^*k*/(1 + 2*ϕ*), which is achieved for *β* = 1, i.e., for a circular motion of the cilia tips. The case *β* = 0, together with a finite value of *ε* = *a*/*h* corresponds to the limit of the flat horizontal motion of the cilia tips. The dimensionless fluid velocity at the cilia wall in this situation is:

$$u\left(0\right)=\frac{\varepsilon^{2}k}{2(1+2\phi )}$$ (65)

One would think that the limit case *β* = 0 would lead to a vanishing steady velocity of the fluid, since all cilia undergo the same oscillatory motion, with a left/right symmetry. What we see here is that the metachronal wave (antiplectic, i.e. going backwards for *k* > 0) breaks this symmetry and leads to a positive steady contribution to the fluid velocity. When *k* = 0 (no wave), one recovers a pure oscillatory motion of the fluid, with no net fluid motion.

Finally, the limit *β* → +∞ corresponds to a flat vertical motion of the cilia tips. Since *ε* = *a*/*h* is the adimensioned horizontal beating amplitude, the limit has to be taken with *ε* → 0, *βε* remaining constant as the adimensioned vertical amplitude *b*/*h*. The horizontal component of the steady velocity is then:

$$u\left(0\right)=\lim_{\beta \to +\infty }\frac{\varepsilon^{2}}{2}\;(\frac{1+2\beta -\beta^{2}}{1+2\phi })\;k=-\frac{b^{2}k}{2h^{2}\left(1+2\phi \right)}$$ (66)

Interestingly, the metachronal wave also breaks the left/right symmetry in this case, but in the opposite direction. In summary, the existence of an antiplectic metachronal wave triggers a net fluid motion along *Ox*, in the positive direction for smaller values of *β* and in the opposite direction for larger values of *β*, the critical value *β*_*c*_ being obtained when (*β*_*c*_ − 1)^2^ = 2 (according to Eq 64), i.e., for $\beta_{c}=1+\sqrt{2}\approx 2.41$. In realistic situations, the trajectories of cilia tips are very flat, so that one is always in the case of small values of *β*: the fluid steady motion occurs always in the direction opposite to the metachronal wave propagation.

### Modeling the micro-bead motion

Once the oscillatory velocity field $\vec{U}=\left(u,v\right)$ is computed, the trajectories and the crossing times of micro-beads in this field are calculated by solving their equation of motion:

$$m\;\frac{d{\vec{U}}_{b}^{*}}{dt^{*}}={\vec{F}}_{\text{drag}}\;,$$ (67)

where *m* is the individual mass of the micro-bead, ${\vec{U}}_{b}^{*}$ its velocity, and ${\vec{F}}_{\text{drag}}$ is the drag force applied on the bead by the fluid flow. Assuming a spherical shape for the bead and small speed differences between the bead and the fluid, the drag force takes the Stokes expression

$${\vec{F}}_{\text{drag}}=-6\pi R\mu \;({\vec{U}}_{b}^{*}-{\vec{U}}^{*})\;,$$ (68)

where *R* stands for the bead diameter. Using the previously defined space and time units *h* and *ω*^−1^, the adimensioned version of Eq 67 reads:

$$\frac{d{\vec{U}}_{b}}{dt}=\frac{\vec{U}-{\vec{U}}_{b}}{St_{k}}\;\text{with}\;\;St_{k}=\frac{m\omega }{6\pi R\mu }$$ (69)

*St*_*k*_ is the bead Stokes number, which characterizes the effective inertia of the bead in the fluid flow. For spherical particles of radius *R* and density *ρ*_*b*_, this number also reads:

$$St_{k}=\frac{\frac{4}{3}\pi R^{3}\rho_{b}\omega }{6\pi R\mu }=\frac{2}{9}\frac{R^{2}\rho_{b}\omega }{\mu }$$ (70)

The micro-beads are about 4.5 μm diameter, and made out of polystyrene of density *ρ*_*b*_ of order 1 g.cm^−3^. At 10 Hz in water, the corresponding Stokes number is about 10^−4^, which means that the micro-beads can be considered as massless tracers. Their velocity can be assumed to be permanently equal to the fluid velocity at the same location.

For each micro-bead entering the simulation window at *x* = 0 and a given altitude *y*_0_, the effective speed is computed as:

$$V_{\text{eff}}\left(y_{0}\right)=\frac{L}{\tau (y_{0})}=\frac{L}{\oint_{T_{y_{0}}}dt}=\frac{L}{\oint_{T_{y_{0}}}\frac{ds}{\|\vec{U}\left(s\right)\|}}\;,$$ (71)

where *τ*(*y*_0_) is the crossing time of the micro-bead entering at (0, *y*_0_), and $T_{y_{0}}$ is the trajectory followed by this micro-bead. During each elementary step of this trajectory, the infinitesimal duration is $dt=ds/\|\vec{U}\left(s\right)\|$, $\vec{U}$ being the fluid velocity at curvilinear abscissa *s* of the trajectory. The effective speed $V_{\text{eff}}$ corresponds to the quantity measured in our experiments.

## Results

### Simulation of micro-bead motions

**Fig 3:**
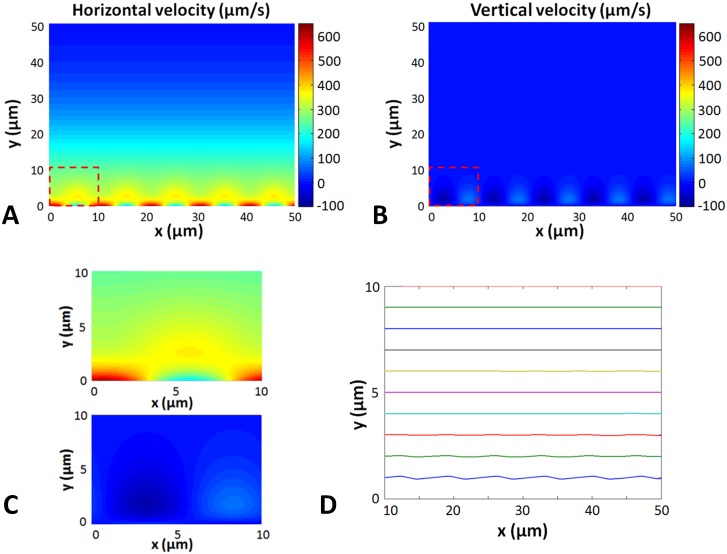
Numerical simulation of the velocity field and micro-bead trajectories. (A) Color view of the horizontal velocity field. The dashed square represents the zoomed-in area represented in Fig 3C. The beating parameters are: CBF = 10 Hz, CBA = 8 μm, λ = 10 μm, Φ = 0, *h* = 50 μm). (B) Color view of the vertical velocity field. The dashed square represents the zoomed-in area represented in Fig 3C. (C) Zoomed in horizontal (top frame) and vertical (bottom frame) velocity field. (D) Particle trajectories for several insertion points. The particles enter the fluid flow on the left side of the window and travel to the right.

The fluid velocity field is solved using the Fourier transform decomposition exposed earlier. This field is periodic both in space and time. Fig 3A and 3B displays the horizontal and vertical components of the fluid velocity, respectively, while Fig 3D presents the micro-bead trajectories in this fluid, simulated by numerically solving Eq 69. One can observe that the beads follow slightly wavy trajectories when close to the cilia wall, but that these trajectories become almost perfect straight lines when moving away from the wall farther than a cilia length. This means that the transit time of a micro-bead across the simulation window is dominated by the steady part of the flow field.

**Fig 4:**
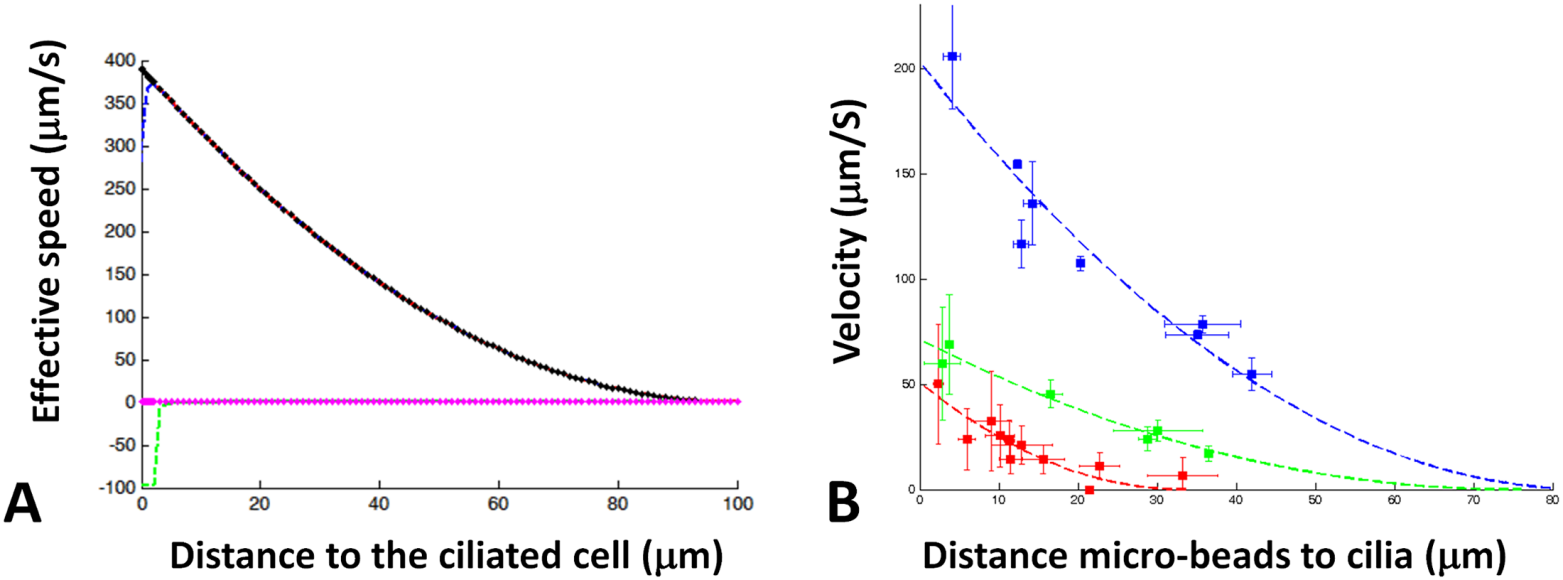
Parabolic velocity profile. (A) Contribution of the different order of the micro-beads velocity The blue dashed line represent the effective velocity (in μm.s^-1^). Green dashed line represent the contribution of the first order of the fluid velocity. The red dashed line represented the contribution of the second order. Black points are the contribution of the parabolic term of the second order. Magenta points are the contribution of the exponential function. Ciliary beating parameters are the following: CBF = 10.0 Hz, CBA = 8.0 μm, λ = 10 μm, *ϕ* = 0 and *h* = 100 μm. (B) Examples of parabolic fitting on microbead velocity measurements on 3 different ciliated edges (see companion paper [11]).

Fig 4A displays the various contributions to the flow profile in the *y*-direction for typical values of the input parameters CBF, CBA, λ, *ϕ*, and *h*. One observes that the total bead velocity (blue line) is essentially dominated by the steady parabolic contribution (black line) determined by the coefficients *A*_2_, *B*_2_, and *C*_2_ in Eq 57. The oscillatory part coming from the first order in *ε* (green line) brings a significant contribution only very close to the ciliated edge, and the exponential contribution in the steady part is negligible everywhere. Consequently, measuring the bead velocity as function of the distance to the ciliated edge essentially amounts to measuring the steady parabolic profile of the flow. Fie 4B shows the excellent agreement between the model and the actual measurements performed on different ciliated edges (here 3 samples from 3 different subjects are presented).

### The ciliary beating efficiency index

The horizontal component of the steady pressure gradient $p_{x}^{\left(s\right)}$ (see Eq 48), applies a force on the fluid directed negatively along O*x*. This force is exactly compensated by the force exerted by the cilia on the fluid, proportional to the dimensionless shear stress ∂*u*/∂*y* (see S1 File for the detailed force balance between the cilia and the fluid). The dimensionless steady force applied by the cilia to a volume fluid of length 2*π*/*k* (the dimensionless wavelength) in the O*x* direction and dimensionless thickness *H*/*h* in the O*z* direction thus reads:

$$F_{w}^{\left(s\right)}=-\frac{H}{h}\frac{2\pi }{k}\frac{\partial u^{\left(s\right)}}{\partial y}{|}_{\left(y=0\right)}=-\frac{\lambda H}{h^{2}}\frac{\partial u^{\left(s\right)}}{\partial y}{|}_{\left(y=0\right)}$$ (72)

Using the parabolic velocity profile *u*^(*s*)^(*y*) = *u*(0)(*y* − 1)^2^ found in Eq 62, and putting back dimensional quantities finally leads to the expression of the local shear stress *τ*_*w*_ applied to the fluid by the cilia:

$$\tau_{w}=\frac{\left(\mu \omega h^{2}\right)F_{w}^{\left(s\right)}}{\lambda H}=\frac{\mu \omega h^{2}}{\lambda H}\frac{\lambda H}{h^{2}}2u\left(0\right)=\frac{2\mu U_{0}}{h}$$ (73)

where *U*_0_ is the velocity extrapolated at *y* = 0 from the measurements of microbead velocities above the cilia. This shear stress characterizes the momentum transfer between cilia and the surrounding fluid. Since *h* is also directly measured by fitting the microbead velocity profile with a parabolic profile, it means that *τ*_*w*_ can be directly deduced using this microbead tracking technique. Consequently, we propose this shear stress as an index for assessing the efficiency of the ciliary beating. One has to stress that we do not intend to reproduce in this model the *in vivo* condition. The shear stress measured using this micro-bead velocity technique is not assumed to be identical to the shear stress experienced by mucus in the pulmonary airways. It is only a way to assess of the ability of the ciliated edge to transfer momentum into the surrounding fluid, thus defining a usable clinical index.

Finally, the extrapolated velocity at the wall can be compared to the one predicted from Eq 64:

$$U_{0}=h\omega \;u\left(0\right)=h\omega \frac{a^{2}}{2h^{2}}\;(\frac{1+2\beta -\beta^{2}}{1+2\phi })\;\frac{2\pi h}{\lambda }=\frac{\pi a^{2}\omega }{\lambda }\;(\frac{1+2\beta -\beta^{2}}{1+2\frac{\phi^{*}}{h}})$$ (74)

The cilium beating amplitude *a* (CBF), the the cilium beating frequency *ω*/2*π* (CBF), and the metachronal wavelength λ are measured directly by microscopic measurements on the cilia, while *h* is extracted from the parabolic fitting of the microbead velocity profile. In the approximation of a flat beating (*β* = 0), this set of measurements therefore provides a way to assess ϕ*, the equivalent sliding length introduced in the boundary condition of Eq 18.

## Discussion

In human airways, the coordinated motion of the cilia covering the epithelium induces a complex displacement of a double layer consisting in a bottom periciliary layer (PCL) and a top mucus layer. Both layers have similar thicknesses, about 5 to 10 μm, but very different viscosities. The situation presented in this paper is however very different from the *in vivo* condition. It reproduces in fact the *ex vivo* experiments performed on a few ciliated cells obtained from nasal brushing. In these experiments, clusters formed of a few ciliated cells are immersed in water, and observed between two microscope slides by high speed video-microscopy. The ciliated edges are therefore lying horizontally in the simulation plane (O*x*, O*y*), which is perpendicular to the optical axis O*z* of the microscope.

The first and foremost difference lies in the fact that the fluid surrounding the cilia is now essentially water, i.e., a homogeneous Newtonian fluid. Secondly, unlike the in vivo situation where mucus is propelled only by the forward trajectory of the cilia tips, here both forward and backward parts of the stroke cycle contribute to the fluid motion. Consequently, as expressed through the mathematical model developed here, the oscillatory motion of the cilia induces at first order in the amplitude *a* an oscillatory motion of the fluid, and at second order a net steady component that pushes the fluid in the direction of the cilia beating. The net motion of the fluid originates from the phase shift between neighboring cilia tips, which breaks the left/right symmetry. More precisely, it creates an unbalance on the envelope between the respective densities of tips moving forward and those moving backwards. This unbalance appears in the second term of Eq 9. One can also note that the direction of the net fluid motion does not depend on the sense of rotation of the tips (clockwise or counterclockwise), but only the direction of the metachronal wave: the fluid is propelled in the direction opposite to the metachronal wave. This property due to the antiplectic nature of the wave might have an interesting consequence: Although the forcing mechanism on the fluid is very different in the presence of mucus (the tips of the cilia enter the mucus layer and push it forward), the fluid covering the cilia would still be pushed in the same direction if the mucus viscosity was significantly lowered or if the mucus would disappear.

In the *ex vivo* experiments, the Womersley number *α* is close to 0.4 while the dimensionless wave vector *k* = 2*πh*/λ is at least about 10 and most of the time much larger than that (*h* is comprised between 30 μm and 140 μm in the experiments, see [11]). This means that the contribution of the convective acceleration to momentum conservation is very small in all cases, and that in first approximation, the system can be understood using a linear Stokes equation point of view. The steady and oscillatory contributions to the wall velocity decouple and induce independent and additive steady and oscillatory contributions to the flow field, respectively. Regarding micro-bead velocity far from the ciliated edge, the system then behaves as an equivalent stationary system with an effective wall velocity *U*_*w*_ = *ε*^2^*k*/2.

At a distance larger than a fraction of the metachronal wavelength from the ciliated edge (the wavelength λ is of order 10–20 μm in all experiments, see [11]), the oscillatory behavior of the flow field vanishes and only the net steady horizontal flow remains. The microbead tracking technique therefore probes this steady part of the flow field, which exhibits a parabolic profile along *y*. The steady contribution to the dimensionless horizontal wall velocity is *ε*^2^*k*/2 (see Eq 11). In the case of flat beating (*β* = 0) and no slip boundary condition at the cilia wall (*ϕ* = 0), this value is exactly the steady fluid velocity at the wall expressed in Eq 65. As a consequence, it also corresponds to the extrapolated micro-bead velocity at the wall. Our model shows that the synchronized elliptic motion of the cilia generates a correction to this velocity by a factor (1 + 2*β* − *β*^2^) (Eq 64). In addition, an imperfect momentum transfer between the cilia and the fluid (*ϕ* > 0) reduces this velocity.

Introducing a partial slip at the cilia wall, through a Robin type boundary condition with an effective sliding length ϕ* = *hϕ* (Eq 18), reduces the fluid velocity by a factor (1 + 2*ϕ*), a result that is also obtained in a purely steady model for a parabolic profile. As shown in the companion paper [11], this effective sliding length is directly correlated to the average density of cilia. This is very consistent with the use of this type of boundary condition in fluid flow next to porous flow, the pore openings corresponding to the empty space between cilia. In clinical application of this model, it is expected that *ϕ* will not be a fitting parameter but predicted by the mere measurement of the cilia density.

### Model limitations

The model proposed in this article relies on several assumptions, hence has a few limitations that we examine now.

First, the motion of each individual cilium tip is assumed to be elliptic. Although this is a generally accepted hypothesis, microscopic imaging of the actual motion of the cilium shows a more complicated trajectory [34]. In particular, the path followed by the tip on the backward trajectory seems to be closer to the forward path than for an elliptic motion. However, we have seen that the net steady motion of the fluid is essentially generated by the metachronal wave. This discrepancy of the trajectory should be accounted for through a change of the parameter *β* representing the ellipse eccentricity. One also has to stress that, since our model reproduces an experimental setup in which cilia are surrounded only by water, one might expect a different motion from the one achieved in *in vivo* situations where cilia are beating in a periciliary liquid while their tips are entering the bottom of the mucus layer.

The model also assumes an exact synchronization of the individual cilia motions, generating a metachronal wave of constant velocity. This implies that the phase shift between two cilia is a linear function of the distance separating them along the direction of the metachronal wave propagation. *Ex vivo* experiments carried out in [11] show that this hypothesis is satisfactorily verified at the observational scale of the experiment, i.e., an edge of a cluster containing a few ciliated cells. A deviation from the linear phase shift would disrupt the translational periodicity of the system and alter our solution based on a Fourier decomposition along the horizontal axis. The fluid velocity at the cilia wall computed in our model can thus be considered as an “ideal velocity”, reached for a perfect metachronal wave. Any measured discrepancy with respect to this ideal velocity could be therefore be attributed either to an imperfect momentum transfer (through the effective slip length *ϕ*), or to a perturbed metachronal wave.

In the model, the ciliated edge is supposed to be flat. This assumption is generally valid in real experiments (see [11]). Cases in which cell clusters appear to have a rough, curved, or disrupted ciliated edge are removed from the measurement procedure.

An important assumption lies in the fact that the fluid surrounding the cilia is stagnant at a distance *h* above the edge. The stagnation of the fluid above the cilia is observed experimentally, and originates from the external environment of the measured cell cluster. Various explanations can account for it: it can be due to the presence of other cell clusters at a few hundred microns distance which create a complex fluid flow in the entire system, or by the friction of the fluid on the upper and lower slides, or the appearance of boundary layers. Determining this value would require modeling the full 3D geometry of the system whose geometry is not easily accessible by microscopic observation. The distance *h* therefore is treated as the only fitting parameter of the model (the sliding length *ϕ* being calculated from the measured cilia density).

Finally, the bead velocities are found in the model to be almost always parallel to the cilia wall. In real experiments, a vertical component (i.e., in the direction perpendicular to the wall) might appear, although much smaller than the horizontal component. This is in particular the case when the ciliated edge is not strictly flat, or when the observation window is such that the ciliated edge is on one side of the window and the bead passing on the other side of the same window. The micro-beads are in this situation not set into motion only by the considered ciliated edge, and external influence originating from outside the observation window interfere, contributing to create a more complex fluid flow pattern. Such situations should be discarded from the analysis, either through eye examination or by an automatized procedure.

### Conclusion

We have developed a mathematical model of micro-bead velocity in a fluid set into motion by the periodic beating of a ciliated edge. The cilia wall is represented as a continuous envelope whose motion is calculated from the coordinated movement of all cilia. The boundary condition imposed by the effective wall induces a motion of the fluid above the wall. This motion has two components: one oscillatory, at the spatial and temporal periodicity of the metachronal wave and the cilia beating, respectively, and one steady, oriented along the direction of the wall. The oscillatory component vanishes at a distance of the order of the metachronal wavelength. The steady component extends on a much longer distance and exhibits a parabolic profile in the direction perpendicular to the wall. The parameters governing this profile are the fluid viscosity, the ciliary beating frequency (CBF), the ciliary beating amplitude (CBA), the metachronal wavelength (λ), the cilia density (through the sliding length ϕ*), and the distance from the wall to the region of stagnant fluid (*h*). Polystyrene micro-beads immersed in the fluid act as massless tracers, allowing the measurement of the local fluid velocity, hence the measurement of the velocity profile. All aforementioned parameters can be determined through local measurement by high speed video-microscopy, except for the distance *h* which remains the only fitting parameter of the model extracted from the velocity profile. The velocity extrapolated from the profile at the wall is found to be linearly related to the shear stress exerted by the cilia on the fluid. This shear stress is proposed as a new index for assessing the efficiency of the ciliary beating. One has to stress that this index can be measured from nasal brushing in the clinical setting without any modification of the current clinical procedures. Preliminary tests (see [11]) have already shown that this index has the potential to be a powerful screening test, able to distinguish patients suffering from various alterations of the cilia beating such Primary Ciliary Dyskinesia (PCD).

## Supporting information

S1 File Force balance on a fluid volume. Derivation of shear stress exerted by the cilia motion onto the surrounding fluid.
(PDF)
