# Supplementary material for: A new index for characterizing micro-bead motion in a flow induced by ciliary beating: Part II, modeling
Source: PLoS Comput Biol. 2017 Jul 14;13(7):e1005552. doi: 10.1371/journal.pcbi.1005552 (PMC5510810; doi:10.1371/journal.pcbi.1005552)
Supplement: S1 File — Derivation of shear stress exerted by the cilia motion onto the surrounding fluid. (PDF) [file pcbi.1005552.s001.pdf]

Supporting Information S1

Force balance on a fluid volume

We detail here the momentum balance equation on a volume of fluid and deduce from it the force exerted by the ciliated edge on the fluid. The simulation volume V is defined by:

$$0 \leq x \leq \frac{2\pi}{k} \quad , \quad 0 \leq y \leq 1 \quad , \quad 0 \leq z \leq \frac{H}{h} \ , \tag{1}$$

where $2\pi/k = \lambda/h$ is one adimensioned metachronal wavelength, and H is the width in the z-direction perpendicular to the 2D channel. Since nothing depend on z in the model, one expects H to disappear from the final expressions. The steady contribution of the integral formulation of the momentum conservation applied to this volume V gives:

$$\rho \left[\iiint\limits_V \left(\vec{U} \cdot \vec{\nabla}\right) \vec{U} \ ds \right]^s = \iint\limits_S -P^s \vec{n} \ ds + \iint\limits_S \left(\underline{\sigma}^s \vec{n}\right) ds \tag{2}$$

where $\vec{U} = (u, v, 0)$ is the velocity field, S is the surface enclosing the volume V, $\vec{n}$ is the exiting normal unit vector on S, P^s is the steady component of the pressure, and $\underline{\sigma}^s$ is the steady stress tensor given by:

$$\begin{cases} \sigma^s_{xx} = \sigma^s_{yy} = 0 \\ \sigma^s_{xy} = \sigma^s_{yx} = \dfrac{\partial u^s}{\partial y} \end{cases} \tag{3}$$

Since div $\vec{U} = 0$, the convective acceleration in Eq 2 (the bracketed expression) can be rewritten as

$$\iiint\limits_V \left(\vec{U} \cdot \vec{\nabla}\right) \vec{U} \ ds = \iiint\limits_V \left[\left(\vec{U} \cdot \vec{\nabla}\right) \vec{U} + \left(\text{div } \tilde{\text{U}}\right) \vec{U} \right] ds = \iint\limits_S \left(\vec{U} \cdot \vec{n}\right) \vec{U} \ ds \tag{4}$$

The x component of the above expression vanishes due to the equal and opposite contributions of the $x = 0$ and $x = L$ sides of surface S. Therefore, after projection on the x-axis, Eq 2 reduces to:

$$0 = \iint\limits_{x=0} P^{(s)} dydz - \iint\limits_{x=\frac{2\pi}{k}} P^{(s)} dydz - \iint\limits_{y=0} \frac{\partial u^{(s)}}{\partial y} \ dydz \tag{5}$$

From Eq 48 in the article, we know that the pressure gradient along x, $p_x^{(s)}$, and the steady shear stress $\partial u/\partial y$ do not depend on x. One can therefore assess the above integrals:

$$0 = \frac{H}{h} \left(-\frac{2\pi}{k} \ p_x^{(s)} \right) - \left.\frac{\partial u^{(s)}}{\partial y}\right|_{(y=0)} \frac{H}{h} \frac{2\pi}{k} = \frac{H}{h} \frac{2\pi}{k} \left(-p_x^{(s)} - \left.\frac{\partial u^{(s)}}{\partial y}\right|_{(y=0)} \right) \tag{6}$$

The second term in parentheses corresponds to the total force applied to the fluid by the cilia wall. This force sets the fluid in motion, hence generating the positive pressure gradient p_x which exactly counterbalances the force. The cancellation of these two terms results in a steady motion of the fluid above the ciliated edge. The dimensionless steady force applied to the fluid by the cilia thus reads:

$$F_w^{(s)} = -\frac{H}{h}\frac{2\pi}{k}\frac{\partial u^{(s)}}{\partial y}\Big|_{(y=0)} = -\frac{\lambda H}{h^2}\frac{\partial u^{(s)}}{\partial y}\Big|_{(y=0)} \qquad (7)$$
